# Risk Factors for Disseminated Coccidioidomycosis, United States

**DOI:** 10.3201/eid2302.160505

**Published:** 2017-02

**Authors:** Camila D. Odio, Beatriz E. Marciano, John N. Galgiani, Steven M. Holland

**Affiliations:** National Institutes of Health, Bethesda, Maryland, USA (C.D. Odio, B.E. Marciano, S.M. Holland);; University of Arizona College of Medicine, Tucson, Arizona, USA (J.N. Galgiani)

**Keywords:** Immunodeficiency, Coccidioides, coccidioidomycosis, IL-12/IFN-γ, race, ethnicity, immunosuppression, fungi, interleukin-12, interferon, disseminated coccidioidomycosis, United States

## Abstract

Of 150,000 new coccidioidomycosis infections that occur annually in the United States, ≈1% disseminate; one third of those cases are fatal. Immunocompromised hosts have higher rates of dissemination. We identified 8 patients with disseminated coccidioidomycosis who had defects in the interleukin-12/interferon-γ and STAT3 axes, indicating that these are critical host defense pathways.

Coccidioidomycosis is acquired by inhaling spores of *Coccidioides immitis*. The Centers for Disease Control and Prevention reported 22,401 cases (42.6 cases/100,000 population) in 2011, an increase from 2,265 cases (5.3/100,000) reported in 1998 (*1*). Although *Coccidioides* infection usually produces little illness and results in lifelong immunity, 25%–30% of infections result in protracted but self-limited illness; <1% are complicated by dissemination, which is serious and sometimes fatal (*2*–*4*). Diagnosis and treatment remain challenging, especially in persons with disseminated, severe, or chronic disease, where host immunity plays an important role.

During January–March 2014, we reviewed risk factors for dissemination and summarized all coccidioidomycosis cases in patients with primary immunodeficiency (PID). These cases highlight the importance of the interleukin (IL)–12/interferon (IFN)–γ and signal transducer and activator of transcription 3 (STAT3) pathways in host defense. Dissemination of this typically self-limited pathogen should prompt consideration of underlying host genetic factors.

## Literature Review

Our systematic literature search resulted in 370 case reports of disseminated coccidioidomycosis (DC) published during 1975–2014 (Technical Appendix). DC was defined as a positive culture or histologic finding from a nonpulmonary site. For comparative purposes, patients were further classified by exogenous immunosuppression, pregnancy, or 1 versus >2 extrapulmonary affected sites.

How the host responds to and contains coccidioidomycosis is unclear, but dissemination occurs in 30%–50% of immunosuppressed hosts. Dissemination can be single-site or multisite, is associated with more severe outcomes than disease limited to the respiratory tract, and requires prolonged treatment (*4*). Literature review confirms critical interactions of *Coccidioides* spp. with race/ethnicity, sex, pregnancy, and immune status (Table 1).

**Table 1:** Summary of disseminated coccidioidomycosis cases reported in the literature*

Predisposition/no. sites affected	Sex, no.	Race/ethnicity, %	Age, y, median (range)	Site of disease, %	Survival, %	
Pregnancy, N = 52		Black, 19; white, 14; Hispanic, 11; Asian 3	27 (17–38)	CNS, 18; bone, 5		42
Immunosuppression,† N = 79	M, 59; F, 19	Black, 11; white, 37; Hispanic, 20; Asian, 4	44 (1–83)	CNS, 25; bone, 37		44
Multisite dissemination, N = 100	M, 84; F, 16	Black, 39; white, 17; Hispanic, 13; Asian, 15	36 (1–84)	CNS, 29; bone, 62		72
Single site dissemination, N = 139	M, 115; F, 24	Black, 32; white, 21; Hispanic, 9; Asian, 17	33 (1–73)	CNS, 9; bone, 50		99

*CNS, central nervous system. †Oncologic, n = 8; HIV, n = 12; transplant, n = 24; steroids/immune-modulation, n = 35.

The rate of DC is higher for pregnant woman than for the general population (*5*,*6*). We found dissemination to the nervous system reported in 37% of pregnant women, approximately one third of whom died (https://www.niaid.nih.gov/sites/default/files/HollandTechnicalAppendix.docx). Of total deaths, 75% occurred among women during their third trimester; fetal or infant death occurred in 40% of reported cases. Although one third of pregnant women affected were black, survival did not differ by race.

Despite overall improved survival, immunocompromised persons remain at high risk for fatal DC; the crude mortality rate (CMR) was ≈50% for persons immunocompromised by HIV, cancer, organ transplantation, antigraft rejection medications, antiinflammatory biologicals, or chemotherapy (https://www.niaid.nih.gov/sites/default/files/HollandTechnicalAppendix.docx). CMRs were lower, but still substantial, for patients receiving steroids (https://www.niaid.nih.gov/sites/default/files/HollandTechnicalAppendix.docx). In HIV-infected exogenously immunocompromised patients, coccidioidomycosis was similar to that in persons without HIV/AIDS. CMRs were lower for persons who were able to stop exogenous immunosuppression. Patients with exogenous immunosuppression were 37% white, 20% Hispanic, and 11% black (https://www.niaid.nih.gov/sites/default/files/HollandTechnicalAppendix.docx), similar to the racial/ethnic distribution in DC-endemic areas (California, Arizona: 48% white, 34% Hispanic, 6% black) (*7*,*8*). However, these racial/ethnic differences should be interpreted cautiously because race/ethnicity data were unavailable for 24% of patients with exogenous immunosuppression. Regardless of age, immunosuppressed patients were substantially more likely to have extrapulmonary dissemination, require hospitalization, have progressive infection, or die of coccidioidomycosis.

Most (84%) patients with multisite infection were male, and the number of blacks was double that of any other race (https://www.niaid.nih.gov/sites/default/files/HollandTechnicalAppendix.docx). Additionally, osteomyelitis was more common among blacks (82%) than whites (29%); central nervous system (CNS) infection was more common among whites (59%) than blacks (13%). Hispanics and Asians also had higher rates of osteomyelitis (69% and 60%, respectively) and lower rates of CNS dissemination (38% and 13%, respectively) than whites (https://www.niaid.nih.gov/sites/default/files/HollandTechnicalAppendix.docx). In contrast, among patients with exogenous immunosuppression, differences in rates of osteomyelitis and CNS infections by race were much smaller (44% of blacks with osteomyelitis vs. 24% of whites and 33% of blacks with CNS infection vs. 21% of whites). These data suggest that different immunologic factors that track with race might variably control susceptibility to DC, osteomyelitis, and CNS disease. However, exogenous immunosuppression apparently overrides these racial/ethnic variations.

Consistent with the demographic characteristics of patients with multisite disease, 83% of those with single-site infection were male (https://www.niaid.nih.gov/sites/default/files/HollandTechnicalAppendix.docx). Overall, blacks had more single-site osteomyelitis than whites (64% vs. 41%), and whites had more CNS infection than blacks (17% vs. 2%) (https://www.niaid.nih.gov/sites/default/files/HollandTechnicalAppendix.docx). Thus, despite the lower CMR in single-site disease, racial/ethnic differences in infection site were largely consistent between those with single-site and multisite infection.

Single-site and multisite disease accounted for 86% of extrapulmonary *Coccidioides* infections in blacks and 91% in Asians but for only 56% in whites and 52% in Hispanics. Furthermore, blacks accounted for approximately one third of single-site and multisite infections despite constituting only 6% of the population in coccidioidomycosis-endemic areas. In contrast, only 10% of patients with single-site and multisite disease were Hispanic, even though Hispanics accounted for 35% of the general population in those areas (*7*). The more population-consistent number of blacks with DC among exogenously immunosuppressed persons and the blunting of racial/ethnic differences with exogenous immunosuppression suggest that exogenous immunosuppression overwhelms intrinsic racial/ethnic variations in host defense. Interpretation of these differences is limited by self-identified race/ethnicity, an imprecise surrogate for ancestral genetic origins. Future studies using established ancestral markers will help solidify associations between coccidioidomycosis infection and race/ethnicity.

We identified 8 cases of proven PID with DC (Table 2). Mutations in the IL-12/IFN-γ or STAT3 pathways were diagnosed in PID patients (Technical Appendix Figure); these patients were younger and more racially/ethnically diverse than immunosuppressed single-site and multisite infected groups. All patients with discrete immune defects had prolonged, refractory infection; some were controlled with exogenous IFN-γ. Of the 8 patients, 3 had no relevant prior medical histories, suggesting that discrete mutations in these pathways might go unrecognized until DC develops.

**Table 2:** Patients with disseminated coccidioidomycosis and discrete primary immune deficiencies*

Patient no. (ref)	Age, y/sex	Race/ ethnicity	Medical history	Extrapulmonary disease	Relapse	Method of diagnosis	Genetic findings
1	4/F	White	HIES, recurrent pneumonia and otitis, skin infections, eczema, thrush	Meningitis	No	BAL/CSF cultures	STAT3: heterozygous (c.2137G>A)
2 (*9*)	17/F	Not reported	HIES, *Staphylococcus. aureus* skin and soft tissue infections, recurrent sinus infections, pneumonia	Meningitis, cerebral abscess	No	*Coccidioides* Ab, CSF culture	STAT3: heterozygous (p.T412S)
3 (*10*)	11/M	White	11 mo: *Mycobacterium chelonei* pneumonia 22 y; disseminated *M. kansasii*	Osteomyelits, lymphadenitis	Yes	*Coccidioides* Ab level, lymph node biopsy	IFN-γR1: deficiency (c.818del4fs)
4 (*11*)†	22/F	Palestinian	11 y: *Salmonella* serogroup D lymphadenitis	Diffuse lymphadenitis	Yes	*Coccidioides* IgM and IgG, lymph node biopsy	IL-12Rβ1: homozygous (p.C186Y)
5 (*11*)	6/M	Palestinian	No other significant history	Osteomyelitis, lymphadenitis, nasal lesion,	Yes	Lymph node, nasal lesion, bone biopsies	IL-12Rβ1: homozygous (p.C186Y)
6	15/M	Black	No other significant history	Osteomyelitis, soft tissue	Yes	BAL cultures, bone and soft biopsies	IL-12Rβ2: heterozygous (p.C101Y)
7 (*12*)	17/F	Hispanic	14 y: extensive, persistent tinea capitis and kerion caused by *Trichophyton tonsurans*	Osteomyelitis, soft tissue, cutaneous lesions	Yes	*Coccidioides* Ab, skin biopsy	STAT1: gain of function mutation (p.E353K)
8 (*12*)	9.5/F	White	No other significant history	Osteomyelitis, cerebral lesions, intrathoracic lymphadenitis	Yes	*Coccidioides* Ab	STAT1: gain of function mutation (p.A267V)

*Ab, antibody; BAL, bronchoalveolar lavage; CSF, cerebrospinal fluid; HIES, hyperimmunoglobulin E (Job’s) syndrome; ref, reference. †Patients 4 and 5 are siblings, and their parents are first cousins.

In *Coccidioides*-susceptible mice, exogenous IL-12 is protective, whereas disease in resistant strains is exacerbated by its neutralization (*13*). In vitro, human macrophage killing of phagocytosed *Coccidioides* depends on IL-12/IFN-γ signaling (*14*). Furthermore, peripheral blood mononuclear cells from nonimmune (delayed-type hypersensitivity-negative) donors produce significantly less IFN-γ in response to *Coccidioides* antigens than do such cells from immune (delayed-type hypersensitivity-positive) donors. In vivo, 3 patients with DC improved substantially after therapy with IFN-γ. Immune function studies in 2 of those patients showed blunted IFN-γ–mediated responses (*15*).

The involvement of STAT3 in resistance to *Coccidioides* infection is complex. STAT3 is a critical mediator of IL-23 signaling, a cytokine involved in producing IFN-γ, IL-12, and IL-17, all of which are required for immunity to *Coccidioides* in vivo. It also might be involved downstream of dectin-1, which is required for resistance to *Coccidioides* in mice and induces the phosphorylation of STAT3.

## Conclusions

Risk factors for DC include exogenous immunosuppression (steroids and biologicals), pregnancy, race/ethnicity, and discrete genetic defects. Although racial/ethnic associations with DC were evident in patients without known underlying risks, they were submerged by exogenous immunosuppression.

Functional and genetic studies indicate that the IL-12/IFN-γ axis and STAT3-mediated immunity are central to protection against *Coccidioides*. We identified mutations affecting these pathways in 8 patients with especially severe or refractory DC, some of whom responded to IFN-γ therapy. Younger patients with severe DC or patients whose illness relapses should be considered for genetic screening for discrete primary immune defects. The discrete defects demonstrated here clearly do not account for all occurrences of coccidioidomycosis in the general population but highlight the importance and nature of genetic control.

Coccidioidomycosis is distinguished by its geography and relative virulence in many persons who otherwise appear immunologically competent. Because most persons in whom DC develops are previously healthy, *Coccidioides* most likely exploits a very narrow vulnerability. The demonstration that DC has an underlying genetic predisposition indicates that the advent of newer genetic techniques, such as whole exome/genome sequencing, will inevitably identify coccidioidomycosis-specific genetic factors. These, in turn, should enable us to better understand, preempt and treat coccidioidomycosis.

Technical Appendix Additional methods for study of risk factors for disseminated coccidioidomycosis and IL-12/IFN-γ signaling pathway.
