# Supplementary material for: Risk Factors for Disseminated Coccidioidomycosis, United States
Source: Emerg Infect Dis. 2017 Feb;23(2):308–11. doi: 10.3201/eid2302.160505 (PMC5324825; doi:10.3201/eid2302.160505)
Supplement: Technical Appendix — Additional methods for study of risk factors for disseminated coccidioidomycosis and IL-12/IFN-γ signaling pathway. [file 16-0505-Techapp-s1.pdf]

Article DOI: http://dx.doi.org/10.3201/eid2302.160505

Risk Factors for Disseminated Coccidioidomycosis, United States

Technical Appendix

Methods

We conducted a systematic search of MEDLINE by PubMed (http://www.ncbi.nlm.nih.gov/PubMed), Web of Science, and Scopus (http://Scopus). Search terms were coccidioidomycosis, Coccidioides, coccidioidal, and disseminated coccidioidomycosis, which were used as both free terms and Medical Subject Headings (MeSH) terms. Terms referring to pregnancy and race were combined with "OR" and "AND." Statistical significance was reported where available as reported in the cited reports.

We identified 4,054 citations; after excluding duplicates and focusing on the criteria of "disseminated coccidioidomycosis," we screened 402. Disseminated coccidioidomycosis was defined as a positive culture or histologic finding from a nonpulmonary site. We acquired the following information: journal article citation, study type, age of patients at time of infection, sex, source population, clinical manifestations and outcome. Treatment and mortality data were organized by decade to reflect changes in therapy. We made secondary classifications as follows: 1) exogenous immune suppression (medication-related immune suppression or HIV); 2) pregnancy (included all patients who were pregnant at the time of coccidioidomycosis); 3) multisite disseminated coccidioidomycosis (MDCM, 2 separate sites with coccidioidomycosis outside of the lungs); 4) single-site disseminated coccidioidomycosis (SDCM, 1 site with coccidioidomycosis outside of the lungs); 5) primary immune deficiency (PID, patients with sequenced genetic mutations and coccidioidomycosis). Races were categorized as defined in the published case reports, with the exception of Asian, which included patients described in case reports as East Asian, West Asian, or Filipino; these we specified where possible. We reviewed 370 case reports of disseminated CM published during 1975–2014, comparing disease presentations.

For the clinical cases described, the patients were enrolled in protocols approved by the institutional review board of the National Institute of Allergy and Infectious Diseases, National Institutes of Health. The patients and/or their families provided written informed consent.

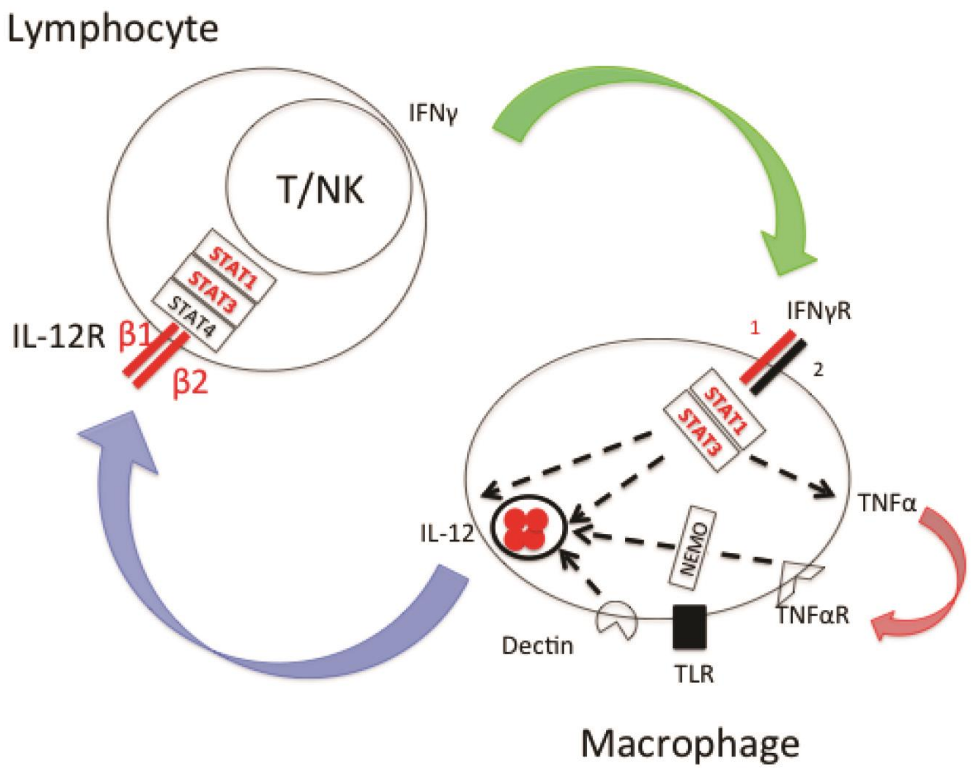

Technical Appendix Figure. IL-12/IFN-γ signaling pathway. Macrophages respond to intracellular bacterial pathogens and dimorphic fungi through the activation of toll-like receptors (TLR), TNF-α receptors, and dectin, among others. This induces the release of IL-12 that binds to its receptors on T/NK cells and stimulates the production of IFN-γ, predominantly through STAT4 signaling, although STAT1 and STAT3 are also engaged. IFN-γ in turn stimulates macrophages through its receptors, using STAT1 and STAT3 to induce TNF, IL-12 and reactive oxygen species to kill intracellular pathogens. Mutations in receptors and intracellular signaling molecules along this pathway increase the susceptibility to mycobacteria, intracellular bacteria, and dimorphic fungi.
